# Lymphome B à grandes cellules primitif du médiastin chez la femme: à propos de cinq cas

**DOI:** 10.11604/pamj.2016.24.181.9906

**Published:** 2016-06-30

**Authors:** Safaa Ouassou, Laila Herrak, Leila Achachi, Fatima Nachite, Kaoutar Znati, Mustapha El Ftouh

**Affiliations:** 1 Service de Pneumologie, Hôpital Ibn Sina, CHU Rabat, Maroc; 2 Laboratoire d’Anatomo-pathologie, Hôpital Ibn Sina, CHU Rabat, Maroc

**Keywords:** Lymphome B primitif du médiastin, lymphome médiastinal, lymphome non-Hodgkinien, médiastin, femme, Primary mediastinal large B-cell lymphoma, mediastinal lymphoma, non-Hodgkin lymphoma, mediastinum, woman

## Abstract

Le lymphome médiastinal primitif à grandes cellules B (LBPM) est un lymphome survenant dans le médiastin antérieur à partir des cellules B de la zone médullaire thymique. Il s’agit d’une entité rare qui présente des particularités tant sur le plan épidémiologique, clinique et évolutif, que sur le plan anatomo-pathologique et immuno-histochimique. Nous rapportons une série de 5 patientes hospitalisées au service de pneumologie de l’hôpital Ibn Sina entre Janvier 2012 et Mai 2016, et chez qui on a retenu le diagnostic de LBPM. L’âge moyen était de 34 ans, le délai moyen de consultation était de 2 mois. Les symptômes rapportés étaient la dyspnée, la douleur thoracique, la toux sèche; deux patientes présentaient un syndrome cave supérieur. Le taux de LDH était élevé chez 4 patientes. L’imagerie thoracique montrait chez les 5 patientes un processus tissulaire médiastinal antérieur. Le diagnostic histologique était posé sur ponction biopsie transpariétale scanno-guidée chez les 5 patientes, et l’apport de l’immunohistochimie était déterminant dans tous les cas. Les patientes ont été adressées à l’institut national d’oncologie pour prise en charge thérapeutique. Le pronostic du LBPM est réservé, il survient volontiers chez des femmes jeunes, ce qui rend d’autant plus nécessaire une thérapeutique agressive afin d’améliorer le taux de survie.

## Abstract (en)

Primary mediastinal large B-cell lymphoma (PMBL) is a lymphoma occurring in the anterior mediastinum starting from the cells B of the thymique medullary zone. This is a rare entity characterized by epidemiological, clinical and evolutionary peculiarities as well as by pathological and immunohistochemical peculiarities. We report a case series of 5 patients with diagnosed PMBL hospitalized in Pulmonology Department of Ibn Sina Hospital between January 2012 and May 2016. The average age was 34 years, the median of consultation time was 2 months. Reported symptoms were dyspnea, chest pain, dry cough; two patients suffered from superior vena cava syndrome. LDH level was high in 4 patients. Thoracic imaging showed an anterior mediastinal tissue processing in 5 patients. Histological diagnosis was based on ultrasound-guided transparietal puncture biopsy in 5 patients. The contribution of immunohistochemistry was decisive in all cases. Patients were sent to the National Institute of Oncology for therapeutic management. PMBL prognosis is reserved, it most commonly occurs in young women, which increases the need of aggressive therapy to improve survival rate.

## Introduction

Le lymphome B à grandes cellules primitif du médiastin (LBPM) est un lymphome survenant dans le médiastin antérieur, plus particulièrement à partir des cellules B de la zone médullaire thymique. Il s’agit d’une entité rare, constituant moins de 3% de tous les lymphomes non hodgkiniens [1], et environ 5% des lymphomes agressifs de l’adulte [2]. Il a été reconnu comme une entité clinico-pathologique distincte des autres sous-groupes du lymphome B diffus à grandes cellules dans la classification OMS 2008 des tumeurs du tissu hématopoïétique et lymphoïde, présentant des particularités tant sur le plan épidémiologique, clinique et évolutif, que sur le plan anatomo-pathologique et immuno-histochimique.

## Méthodes

Nous rapportons une série de 5 patientes hospitalisées au service de pneumologie de l’hôpital Ibn Sina entre Janvier 2012 et Mai 2016, et chez qui on a retenu le diagnostic de lymphome B à grandes cellules primitif du médiastin sur les résultats anatomo-pathologiques, immunohistochimiques, et phénotypiques.

## Résultats

**Figure 1:**
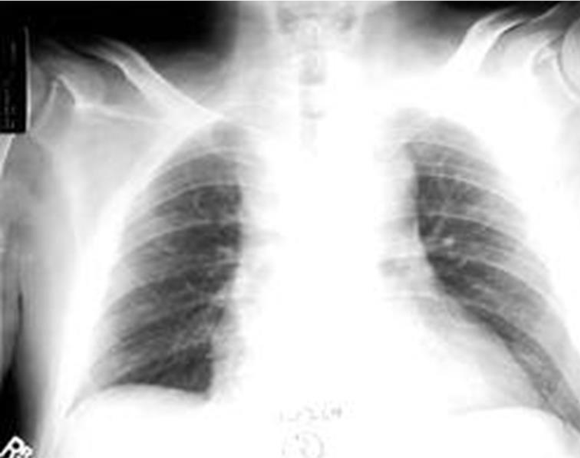
Important élargissement médiastinal (radiographie thoracique de face)

**Figure 2:**
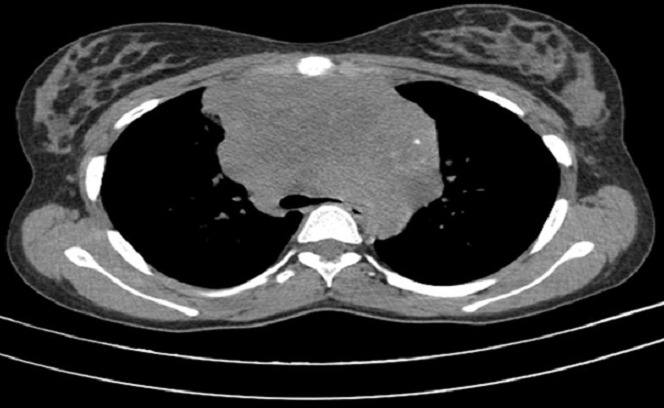
Processus médiastinal antérieur renfermant des calcifications, avec effet de masse important sur la trachée (coupe TDM en fenêtre médiastinale)

**Figure 3:**
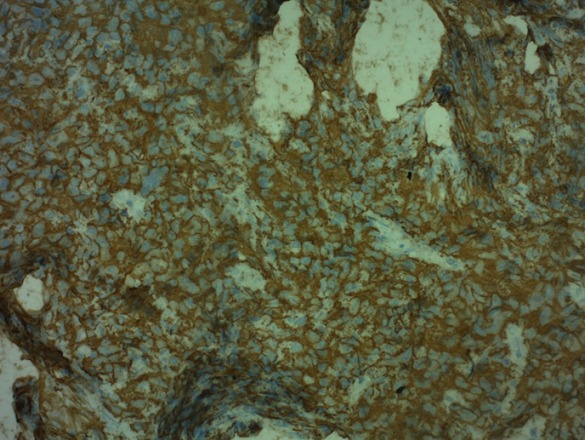
Expression diffuse par les cellules tumorales de l’anticorps anti-CD20 (immunohistochimie, ×20)

L’âge moyen de nos patientes était de 34 ans; le délai moyen de consultation par rapport au début de la symptomatologie clinique était de 2 mois. Les symptômes rapportés étaient la dyspnée dans 3 cas (60%), la douleur thoracique dans 4 cas (80%), et la toux sèche dans 3 cas (60%). Deux patientes présentaient un syndrome cave supérieur (40%). Chez toutes nos patientes, l’examen clinique ne retrouvait ni adénopathie périphérique, ni hépatosplénomégalie. Le taux de LDH était élevé chez 4 patientes (80%), avec un taux moyen de 530 UI/L. L’imagerie thoracique montrait chez les 4 patientes un processus tissulaire médiastinal antérieur (100%), dont la taille était supérieure à 10 cm dans 3 cas (60%), associé à d’énormes adénopathies médiastinales chez 1 patiente (20%), à des nodules pulmonaires chez 1 patiente (20%), et à un épanchement pleural chez 3 patientes (60%) (Figure 1, Figure 2). La fibroscopie bronchique n’a pas montré d’anomalie endobronchique dans tous les cas. Le diagnostic histologique était posé sur biopsie transpariétale scanno-guidée chez les 5 patientes (100%). L’analyse anatomo-pathologique des prélèvements a objectivé une prolifération de cellules lymphoïdes de moyenne à grande taille, à cytoplasme clair et abondant, avec des noyaux volumineux, nucléolés (Figure 2), au sein d’une fibrose tissulaire; l’apport de l’immunohistochimie et du phénotypage (CD20+, CD23+, CD30+, CD15-) (Figure 3), était déterminant dans tous les cas pour faire la différence avec un lymphome hodgkinien dans sa forme scléronodulaire. Chez une patiente, le bilan d’extension avait révélé des localisations thyroïdienne, mammaire et hépatique; chez les 4 autres patientes, le bilan d’extension n’a pas révélé d’autres localisations. Le caractère primitif de cette localisation tumorale médiastinale a alors été retenu. Les patientes ont été adressées à l’institut national d’oncologie pour prise en charge thérapeutique.

## Discussion

Les lymphomes médiastinaux sont très principalement des lymphomes agressifs touchant des sujets jeunes. Trois entités résument la quasi-totalité de cette pathologie: le lymphome lymphoblastique T, le lymphome B à grandes cellules médiastinal et le lymphome hodgkinien classique scléronodulaire [3]. Les lymphomes B à grandes cellules primitifs du médiastin (LBPM) ont longtemps été considérés comme une variante des formes classiques de lymphomes B diffus à grandes cellules. En pratique, tout les opposait sur les plans cliniques, histopathologiques, génétiques et évolutifs, si bien que la classification des lymphomes de l’OMS de 2008 les distingue désormais comme une entité à part [4, 5]. Les LBPM se développent probablement à partir des lymphocytes B thymiques, cette hypothèse est confortée par l’observation, sur le plan génétique, d’une surexpression caractéristique du gène myelin and lymphocyte protein (MAL) dans 70% des cas de LBPM, protéine également exprimée dans les lymphocytes B de la médullaire thymique [6]. Par rapport au lymphome B diffus à grandes cellules, le LBPM survient chez des sujets plus jeunes, 30 à 40 ans [2], avec une prédominance féminine, un taux élevé de LDH, et une extension intra-thoracique fréquente aux organes de voisinage comme la plèvre, le péricarde et le parenchyme pulmonaire [7]. Comme la tumeur est souvent volumineuse, supérieure à 10 cm, les sujets se présentent avec un syndrome cave supérieur ou des symptômes en rapport avec un syndrome médiastinal (toux, dyspnée) [8]. Les données de notre série rejoignent en ceci parfaitement celles de la littérature, avec un âge moyen de 34 ans, des symptômes faits de toux, dyspnée, douleurs thoraciques, et syndrome cave supérieur, et un taux de LDH élevé dans 80% des cas. L’extension aux organes de voisinage est également retrouvée dans notre série, avec une atteinte parenchymateuse pulmonaire dans 20% des cas, et une extension pleurale dans 60% des cas. Sur le plan anatomo-pathologique, l’aspect typique est celui de plages diffuses marquées par la prolifération de grandes cellules rondes au cytoplasme clair, associée à une fibrose, soit épaisse, hyaline, soit pénicillée, compartimentant la population tumorale [1]. L’étude immunohistochimique a un intérêt majeur pour poser le diagnostic et éliminer les autres tumeurs lymphoïdes et/ou fibrosantes. Il s’agit de cellules B exprimant CD19, CD20, CD22 et CD79a, CD23 et, pour 69% d’entre eux, CD30, mais pas CD15 [9, 10]. Une étude pakistanaise a démontré que le LCA (Leucocyte Cell Antigen) est le seul marqueur permettant de différencier définitivement un LBPM d’un cHL (classique Lymphome de Hodgkin), lorsque les caractéristiques de ces deux entités se chevauchent par ailleurs. Ce marquage n’est malheureusement pas disponible en routine dans notre laboratoire d’anatomo-pathologie [11]. Depuis sa publication en 1993, l’index pronostique international (IPI) est l’outil utilisé pour la prise en charge thérapeutique des patients porteurs de lymphomes B à grandes cellules [12]. Néanmoins, l’IPI est probablement non discriminatif chez les patients atteints de LBPM. En effet, la maladie concerne des patients jeunes avec une tumeur souvent limitée au médiastin. Ainsi, malgré la gravité de la pathologie, le score IPI est faible pour plus de la moitié des patients et ne reflète pas la réalité de la situation [13]. Une étude canadienne a montré que l’âge supérieur à 40 ans, le taux de LDH supérieur à deux fois la normale et le Performans Status supérieur ou égal à deux étaient associé à une évolution défavorable [14]. Le LBPM est une entité à la fois rare et nouvellement reconnue, il existe donc très peu de données prospectives et un manque d’études randomisées sur les protocoles thérapeutiques, et les controverses sont nombreuses autour de l’approche thérapeutique optimale. Chez ces jeunes patients, il est capital de prendre en considération dans les choix thérapeutiques les effets secondaires à long terme. Il est important de choisir un traitement de première ligne optimal, d’autant plus que les traitements au moment de la rechute donnent des résultats médiocres [14, 15]. Les progrès thérapeutiques donnent des résultats encourageants avec l’apport des anticorps monoclonaux (Rituximab), et l’utilisation de la consolidation par haute dose de chimiothérapie chez les patients les plus graves. Le protocole DA-R-EPOCH (dose-adjusted Rituximab, Etoposide, Prednisone, Vincristine, Cyclophosphamide, Doxorubicin) utilisé dans une étude rétrospective en phase II a montré des résultats satisfaisants, avec un taux de réponse complète de 94%, et une survie sans évènement de 93% à 3 ans. [16]. La place de la radiothérapie est loin d’être clairement définie, certaines études suggèrent qu’elle aurait surtout un intérêt dans les cas de rémission partielle, qui peuvent devenir complète après radiothérapie. L’imagerie par TEP-FDG est un outil d’évaluation qui peut permettre de déceler les maladies réfractaires de façon précoce, permettant ainsi de poser l’indication d’une radiothérapie de consolidation [17]. Dans notre travail, nous n’avons pas pu évaluer la réponse thérapeutique, ni le suivi au long cours, puisque nos patientes ont été adressées à l’institut national d’oncologie pour la prise en charge thérapeutique.

## Conclusion

Si le pronostic du LBPM était réservé, les progrès réalisés en matière de phénotypage permettent désormais de définir des profils moléculaires bien précis, permettant ainsi une approche thérapeutique de plus en plus ciblée, en phase avec l’ère de la médecine personnalisée. Il n’en reste pas moins que cette maladie survient volontiers chez des femmes jeunes, ce qui rend d’autant plus nécessaire des études randomisées prospectives pour mettre en place des protocoles visant à améliorer la survie, tout en minimisant les effets secondaires à long terme.

### Etat des connaissances actuelles sur le sujet

- Les lymphomes B médiatisnaux primitifs se développent probablement à partir des lymphocytes B thymiques;
- Les lymphomes B médiastinaux primitifs sont des lymphomes agressifs touchant des sujets jeunes;
- Les lymphomes B médiastinaux primitifs présentent des particularités tant sur le plan épidémiologique, clinique et évolutif, que sur le plan anatomo-pathologique et immuno-histochimique.

### Contribution de notre étude à la connaissance

- Particularités épidémiologiques (prédominance féminine);
- Particularités anatomo-pathologiques et immuno-histochimiques;
- Approche thérapeutique avec discussion de l’apport de chaque modalité thérapeutique, en tenant compte du rapport bénéfice/risque.
